# Predictors, clinical impact, and management strategies for conduction abnormalities after transcatheter aortic valve replacement: an updated review

**DOI:** 10.3389/fcvm.2024.1370244

**Published:** 2024-04-08

**Authors:** Qingyun Yu, Qingan Fu, Yunlei Xia, Yanqing Wu

**Affiliations:** Department of Cardiology, The Second Affiliated Hospital of Nanchang University, Nanchang, China

**Keywords:** transcatheter aortic valve replacement, conduction abnormalities, pacemaker implantation, predictors, prognosis, management

## Abstract

Transcatheter aortic valve replacement (TAVR) has increasingly become a safe, feasible, and widely accepted alternative surgical treatment for patients with severe symptomatic aortic stenosis. However, the incidence of conduction abnormalities associated with TAVR, including left bundle branch block (LBBB) and high-degree atrioventricular block (HAVB), remains high and is often correlated with risk factors such as the severity of valvular calcification, preexisting conditions in patients, and procedural factors. The existing research results on the impact of post-TAVR conduction abnormalities and permanent pacemaker (PPM) requirements on prognosis, including all-cause mortality and rehospitalization, remain contradictory, with varied management strategies for post-TAVR conduction system diseases across different institutions. This review integrates the latest research in the field, offering a comprehensive discussion of the mechanisms, risk factors, consequences, and management of post-TAVR conduction abnormalities. This study provides insights into optimizing patient prognosis and explores the potential of novel strategies, such as conduction system pacing, to minimize the risk of adverse clinical outcomes.

## 1 Introduction

Aortic stenosis (AS), a prevalent cardiac valve disease, is becoming increasingly common due to the increase in life expectancy and the growth of the elderly population (1, 2). Over the past two decades, transcatheter aortic valve replacement (TAVR) has emerged as a minimally invasive alternative, especially for patients with severe symptomatic AS and a high risk for surgical aortic valve replacement (SAVR) (1, 3, 4). Over time, improved surgical techniques, cumulative experience, updated transcatheter heart valve (THV) designs, and better patient selection have collectively enhanced surgical safety, effectively contributing to reductions in perioperative mortality and procedural complication rates (5, 6). However, there is still a relatively greater incidence of cardiac conduction abnormalities after TAVR than after SAVR, and this trend differs from the decreasing incidence of other postoperative complications (7, 8). In particular, the two most frequent types are new-onset left bundle branch block (LBBB) and high-grade atrioventricular block (HAVB), which necessitate permanent pacemaker implantation (PPI) (9, 10). The incidence of conduction abnormalities and PPI post-TAVR generally varies somewhat depending on the THV system used, and we exemplify in Table 1 the incidence of new PPI within 30 days after TAVR using the different THVs. Some of the current findings have tended to show that balloon-expandable valves are more effective in reducing the incidence of post-TAVR conduction abnormalities and permanent pacemaker implantation events than self-expandable valves, but the differences between generations of THVs are not significant in this regard (19, 28–30).

**Table 1:** Incidence of new PPI 30-days after TAVR according to device types.

Device types	Specific device types	Studies	Patients (*n*)	Access approach	Valve size (mm)	Incidence of new PPI (%)
Balloon-expandable Edwards Sapien valve	Sapien XT	Schymik G et al. (11)	2,688	Transfemoral (*n* = 1,685)	23 (*n* = 1,135)	9.5
				Transapical (*n* = 894)	26 (*n* = 1,305)	
				Subclavian (*n* = 8)	29 (*n* = 235)	
				Transaortic (*n* = 101)		
	Sapien 3	Pellegrini C et al. (12)	849	Transfemoral (*n* = 849)	20 (*n* = 7)	9.7
					23 (*n* = 346)	
					26 (*n* = 312)	
					29 (*n* = 184)	
	Sapien 3 ultras	Saia F et al. (13)	139	Transfemoral (*n* = 139)	20 (*n* = 5)	4.4
					23 (*n* = 60)	
					26 (*n* = 53)	
					29 (*n* = 21)	
Balloon-expandable Meril Lifesciences valve	Myval	García-Gómez M et al. (14)	100	Transfemoral (*n* = 98)	21.5 (*n* = 8)	8.0
				Other (*n* = 2)	23 (*n* = 19)	
					24.5 (*n* = 15)	
					26 (*n* = 27)	
					27.5 (*n* = 16)	
					29 (*n* = 15)	
		Elkoumy A et al. (15)	68	Transfemoral (*n* = 67)	20 (*n* = 7)	8.5
				Other (*n* = 1)	21.5 (*n* = 2)	
					23 (*n* = 20)	
					24.5 (*n* = 5)	
					26 (*n* = 14)	
					27.5 (*n* = 5)	
					29 (*n* = 13)	
					32 (*n* = 2)	
Self-expandable Medtronic CoreValve	Evolut R	Dowling C et al. (16)	217	Iliofemoral (*n* = 198)	34 (*n* = 217)	15.7
				Subclavian (*n* = 16)		
				Direct aortic (*n* = 3)		
	Evolut PRO/PRO+	Manoharan G et al. (17)	629 (PRO)	Iliofemoral (*n* = 610)	23 (*n* = 24)	20.7
					26 (*n* = 192)	
					29 (*n* = 394)	
		Scotti A et al. (18)	1,616 (PRO/PRO+)	Transfemoral (*n* = 1,616)	23 (*n* = 54)	14.8
					26 (*n* = 532)	
					29 (*n* = 817)	
					34 (*n* = 34)	
		Costa G et al. (19)	1,366 (PRO/PRO+)	Transfemoral (*n* = 1,366)	23 (*n* = 17)	17.9
					26 (*n* = 253)	
					29 (*n* = 407)	
					34 (*n* = 6)	
	Evolut FX	Zaid S et al. (20)	226	Transfemoral (*n* = 226)	23 (*n* = 11)	11.9
					29 (*n* = 105)	
					26 (*n* = 60)	
					34 (*n* = 50)	
Self-expandable Boston Scientific valve	Acurate neo	Möllmann H et al. (21)	89	Transfemoral (*n* = 89)	23 (*n* = 34)	10.3
					25 (*n* = 35)	
					27 (*n* = 20)	
	Acurate neo2	Möllmann H et al. (22)	120	NA	21–23 (*n* = 31)	15.0
					23–25 (*n* = 54)	
					25–27 (*n* = 35)	
Self-expandable Abbott valve	Portico	Möllmann H et al. (23)	222	Transfemoral (*n* = 222)	23 (*n* = 50)	13.5
					25 (*n* = 52)	
					27 (*n* = 60)	
					29 (*n* = 60)	
	Navitor	Reardon MJ et al. (24)	260	Transfemoral (*n* = 259)	23 (*n* = 14)	19.0
				Subclavian/axillary (*n* = 1)	25 (*n* = 66)	
					27 (*n* = 103)	
					29 (*n* = 77)	
Mechanically expanded Boston scientific valve	Lotus	Montone RA et al. (25)	225	Transfemoral (*n* = 219)	23 (*n* = 85)	31.8
				Subclavian (*n* = 6)	25 (*n* = 89)	
					27 (*n* = 51)	
		Meredith Am IT et al. (26)	120	Transfemoral (*n* = 120)	23 (NA)	28.6
					27 (NA)	
	Lotus Edge	Armario X et al. (27)	286	Transfemoral (*n* = 282)	23 (*n* = 74)	30.8
					25 (*n* = 105)	
					27 (*n* = 107)	

Extensive research indicates that TAVR-treated patients often experience positive clinical outcomes, a factor anticipated to facilitate the adoption of the procedure for more diverse patient groups, including younger individuals and those with low perioperative risks (8, 31–33), but the potential adverse effects of conduction abnormalities and long-term right ventricular pacing post-TAVR might mitigate its superiority. The aim of this review is to dissect the latest insights into the mechanisms, predictors, and types of conduction abnormalities that occur after TAVR, along with their clinical impacts and management tactics to elucidate the relationships and developmental trends between TAVR and new-onset conduction abnormalities and to ultimately provide more refined management approaches for TAVR.

## 2 The anatomical mechanisms underlying the occurrence of conduction abnormalities after TAVR

With the progressive popularization and advancement of TAVR, researchers begun to increasingly focus on accurately visualizing the anatomy of the aortic valve complex, specifically the cardiac conduction system. This emphasis is crucial for effectively preventing the occurrence of new conduction abnormalities after TAVR in the future. Comprehending the anatomical interplay between the aortic valve complex and the cardiac conduction system is also foundational in the study of new-onset conduction irregularities following TAVR.

**Figure 1:**
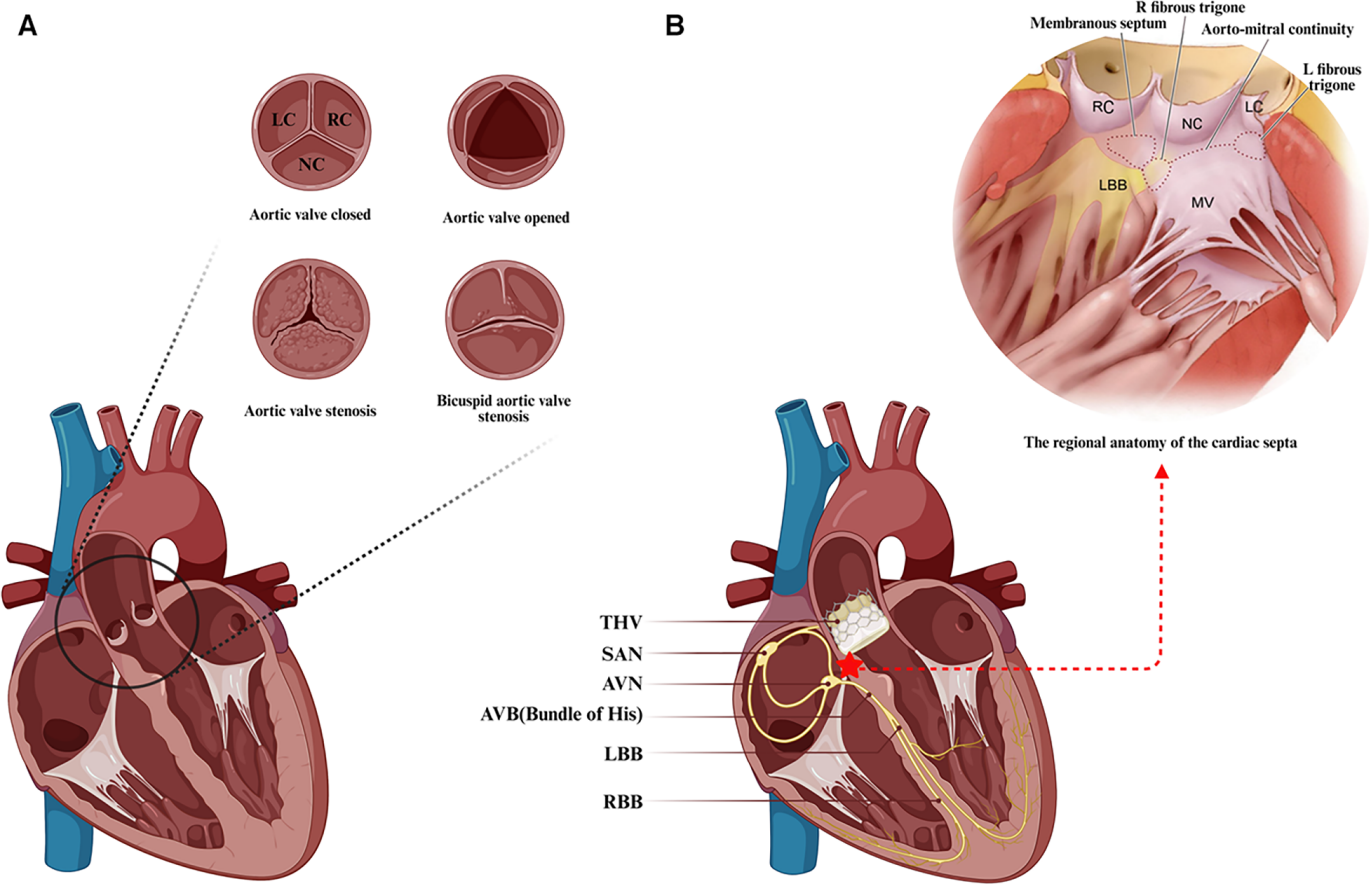
Anatomical features associated with transcatheter aortic valve replacement. (**A**) The anatomy and frequent types of aortic stenosis. (**B**) Positional relationship between the transcatheter aortic valve landing zone and the atrioventricular conduction system. Anatomical schematic of the membranous septal region in the heart from Samuel J et al. (35). LC, left coronary cusp; RC, right coronary cusp; NC, noncoronary cusp; SAN, sinoatrial node; AVN, atrioventricular node; AVB, atrioventricular bundle; LBB, left bundle branch; RBB, right bundle branch; THV, transcatheter aortic valve. (Created with BioRender.com).

The aortic valve, a tri-leaflet structure devoid of vascular supply, is attached to the aortic root via a fibrous annulus. Based on the position of the leaflets relative to the coronary orifices, they are identified as the right coronary cusp (RC), left coronary cusp (LC), and noncoronary cusp (NC) (Figure 1A) (34). Pertaining to the conduction system critical for TAVR, the atrioventricular node (AVN) is located in the right atrium, predominantly at the base of the atrial septum, and is typically identifiable by Koch's triangle, which is composed of Todaro's tendon, the coronary sinus orifice, and the insertion point of the tricuspid valve septal leaflet (36–38). Notably, the vertex of Koch's triangle is proximal to the cardiac central fibrous body. The central fibrous body, comprising the membranous septum (MS) and the right fibrous triangle formed by thickening of the end of the fiber continuity region, separates the subaortic area of the left ventricle from the right atrium and ventricle. It is also a region of the heart where the membranous septum, atrioventricular valve and aortic valve are connected by fibrous continuity (39). The AVN then tapers anteriorly, disengaging from the atrial myocardium and traversing through to the right fibrous triangle, forming the His bundle or atrioventricular bundle. The his bundle ascends inclined anteriorly and superiorly from the posteroinferior to the anterior, crossing the MS and emerging near the aortic root. Then, this bundle forms a bifurcation near the interventricular ridge, with the left branch forming the left bundle branch (LBB) at the interleaflet triangle base between the noncoronary and right coronary sinuses, and the right branch forming the right bundle branch (RBB) just below the medial papillary muscle of the tricuspid valve in the right ventricle (40).

Due to the close anatomical proximity of the conduction system to the distal landing zone of the transcatheter heart valve (THV) located in the left ventricular outflow tract (LVOT), particularly the direct exposure of the His bundle after crossing the membranous septum near the aortic root, the His bundle and the originating portion of the LBB are closely associated with the base of the NC and RC leaflets' apical triangle at the aortic valve (Figure 1B). Technical maneuvers near the aortic root, such as fragmenting the calcified valve or implanting a prosthesis, can pressure surrounding tissues, potentially causing edema, inflammation, ischemia, or hematoma, and consequently new conduction abnormalities, which are a concern during TAVR (38, 41–43). There is correlative evidence linking TAVR-induced conduction abnormalities to calcific aortic stenosis: calcium deposits may also affect the nearby conduction system, and aortic valve stenosis-associated left ventricular (LV) dysfunction may increase the risk of advanced AV block and LBBB (37). Furthermore, the majority of new conduction abnormalities were observed to occur in association with a series of operations prior to the actual implantation of the THV, mainly in the form of postoperative new-onset LBBB. The frequency of these conduction abnormalities during TAVR varies, with the highest likelihood occurring during balloon preexpansion, followed by THV expansion, THV positioning, balloon catheter positioning, and wire crossing of the aortic valve (41, 44). New conduction abnormalities are likely to occur not only during the procedure but also some time afterward, and late-onset new conduction abnormalities warrant further investigation to fully understand their underlying mechanisms (45).

Interestingly, there is notable individual variability in the anatomy of aortic valves and conduction systems. The bileaflet aortic valve (BAV) is the most common aortic valve malformation, often leading to aortic stenosis in younger patients, while unileaflet and quadricuspid aortic valves are rarer anatomical variants (46). Hence, investigating the relevance of conduction abnormalities after TAVR in patients with BAV stenosis is crucial. Anatomical aspects such as the anteroposterior relation of the AVN to Koch's triangle apex, the length of the penetrating portion of the His bundle, the length of the membranous septum, and variations in the position of the AVB and the proximal branching of the LBB show interindividual differences. These interindividual anatomical variations are likely significant factors affecting susceptibility to conduction system injuries. A previous study of 115 autopsies in elderly patients revealed that nearly half of the patients had relatively right-sided AV bundles, approximately 30% had comparatively left-sided AV bundles, and approximately 20% had AV bundles traveling under the septum below the endocardium. In the latter two variants, the location of the AV bundle is more exposed and vulnerable to damage from external forces, especially in patients with a shorter membranous septum, who are at greater risk for conduction abnormalities after TAVR (36, 40).

## 3 Predictors of conduction abnormalities after TAVR

### 3.1 Electrocardiogram-related factors

Numerous studies evaluating the predictors of conduction abnormalities or PPI after TAVR have identified preoperative right bundle branch block (RBBB) as a primary risk factor (47, 48). Additionally first-degree AVB and new-onset LBBB have also been revealed to be significant predictors. Research by Gonska et al. (49) and Keßler et al. (50) independently demonstrated that baseline RBBB and first-degree AVB were predictors of new PPI necessity post-TAVR. In a prospective study, Pavlicek et al. (51) reported that postoperative new-onset LBBB (OR: 15.72; 95% CI: 3.05–81.03; *p* = 0.001) and preoperative bundle-branch block (OR: 11.64; 95% CI: 2.87-47.20; *p* = 0.001) were independent predictors of high-grade atrioventricular block (HAVB) necessitating PPI after TAVR. Data from a Chinese TAVR cohort indicated that new-onset LBBB (*p* = 0.004) and lead I T-wave elevation (*p* = 0.016) were the primary predictors of PPI (52). It was also suggested in some studies that ΔPR (the difference between the postoperative and preoperative PR intervals) was probably an independent predictor of delayed late conduction disorders (≥48 h) after TAVR (53), with ΔPR > 40 ms linked notably to an increased risk of PPI (54). In particular, a study on delayed total atrioventricular block (DT-AVB) morbidity and potential predictors after TAVR highlighted that intraoperative HV interval prolongation (OR: 1.07; 95% CI: 1.02–1.14; *p* = 0.015) and PQ interval prolongation between the next day post-TAVR and baseline (OR: 1.04; 95% CI: 1.01–1.09; *p* = 0.032) might predict the occurrence of DT-AVB (55). Recently, Yagel et al. reported that the R-wave amplitude in the V1 lead of the baseline ECG appeared to predict the onset of HAVB in patients with new-onset LBBB post-TAVR, and patients with HAVB requiring PPI had a significantly lower baseline R-wave amplitude in the V1 lead than those who did not develop HAVB (0.029 ± 0.04 mV vs. 0.11 ± 0.14 mV, *p* = 0.0316) (56).

### 3.2 Anatomy-related factors

Asymmetrical calcification patterns of the aortic valve, as well as heightened calcium burdens in the left ventricular outflow tract or the valve implantation area, are pivotal anatomical factors for predicting PPI necessity post-TAVR. Fujita et al. investigated the impact of aortic valve calcium distribution on atrioventricular block necessitating PPI post-TAVR and revealed that increased calcium in the left coronary cusp (LCC) was an independent risk factor for PPI after TAVR, particularly in patients with LCC calcium loads exceeding 209 mm^3^ (16.7% vs. 2.6%; *p* = 0.003) (57). They hypothesized that this may be related to the significant shift of the balloon and THV with calcium loading from the LCC to the conjunction between the RCC and LCC during the operation. However, Mauri et al. reported that a heightened calcium volume in the LVOT below the LCC or in the RCC was independently correlated with the PPI requirement after TAVR (LVOT LC: *p* = 0.016) (LVOT RC: *p* = 0.005) (58). Another study from the Cedars-Sinai Heart Institute identified elevated calcium volume in the NCC-DLZ area (the region of valve implantation below the NCC) as the most relevant predictor for PPI after TAVR with the Sapien 3 valve (OR: 1.04; 95% CI: 1.02–1.06; *p* < 0.001) (59). Moreover, the membranous septum length has been recognized as a key predictor of post-TAVR conduction abnormalities, with shorter MS lengths seemingly correlating with greater PPI needs (60) Notably, a multicenter study revealed that shorter MS length was an independent predictor of PPI requirements after TAVR for various THV devices, excluding Accurate-THV (Sapien 3: OR: 0.87; 95% CI: 0.79–0.99; *p* < 0.01) (Evolut: OR: 0.91; 95% CI: 0.84–0.98; *p* = 0.03) (61). However, there is currently no evidence of any statistically meaningful effect of THV type on the relationship between MS length and new PPI. Severe mitral annular calcification (MAC) (62), the tapered LVOT (63), the large aortic valve area (AVA) (61), and the high ratio of postoperative to preoperative AVA (64) have also been identified as independent predictors of new PPI after TAVR for the first time in recent studies and are strongly associated with conduction abnormalities after TAVR.

### 3.3 Procedure-related factors

Intraoperative TAVR procedures such as guidewire insertion, balloon dilatation, and valve implantation may inflict direct mechanical injury to the surrounding tissues of the aortic root or induce changes such as inflammation, edema, ischemia, and necrosis. These alterations can compress or impair the conduction system. While no clinical indicators directly assess the extent of intraoperative mechanical injury, other potentially procedure-related risk factors include the THV diameter, THV implantation depth, and THV type. A large meta-analysis revealed that a larger prosthetic valve size (MD: 1.52%; *p* < 0.05) and lower implantation depth (MD: 0.95 mm; *p* < 0.05) were procedural predictors of PPI after TAVR (65). Almeida's et al. retrospective study confirmed that a lower THV implantation depth (OR: 1.16; 95% CI: 1.01–1.33; *p* = 0.035) was an independent predictor of conduction abnormalities after TAVR (66). A larger THV diameter/LVOT diameter was also found to significantly correlate with higher PPI rates after TAVR (67). However, some studies have suggested that both THV implantation depth and oversize are not consistently independent predictors of PPI necessity after TAVR (49). Recently, the differences between membranous septum length and THV implantation depth (ΔMSID) (68) and valve recapture (69) have been shown to predict the onset of post-TAVR conduction system disease, with a ΔMSID < 0 being deemed to be the strongest and most unique modifiable predictor (68). Further studies have shown that both the coronal ΔMSID measured on preoperative CT and the infra-annular ΔMSID measured on postoperative angiography are variable predictors of conduction abnormalities after TAVR, with the coronal ΔMSID being more predictive (95.9% vs. 87.2%; *p* = 0.002) (70). Additionally, the use of self-expanding THV has been identified as a predictive factor, with patients treated with BEV possessing seemingly lower new PPI rates than those receiving SEV (61, 71).

**Figure 2:**
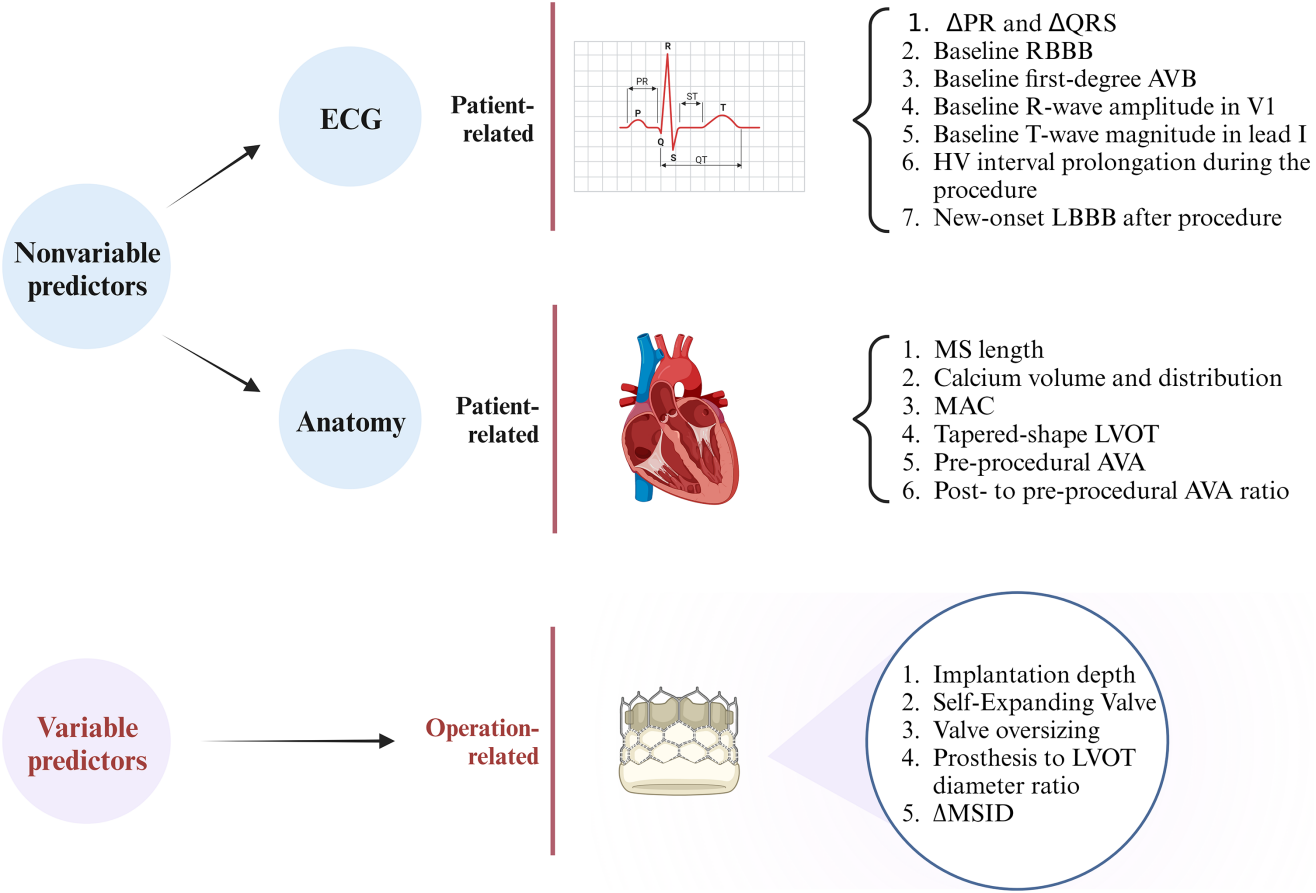
Summary of the predictors of conduction abnormalities after transcatheter aortic valve replacement. ΔPR, difference between postprocedural and preprocedural PR lengths; ΔQRS, difference between postprocedural and preprocedural QRS lengths; RBBB, right bundle branch block; LBBB, left bundle branch block; AVB, atrioventricular block; MS, membranous septum; MAC, mitral annular calcification; LVOT, left ventricular outflow tract; AVA, aortic valve area; ΔMSID, difference between the membranous septum length and implantation depth. (Created with BioRender.com).

Therefore, it seems plausible that the emergence of conduction abnormalities post-TAVR is attributable to a synergistic interplay among patient-specific anatomical characteristics, baseline cardiac electrophysiological features, and procedural interventions. These factors can be broadly categorized into variable and nonvariable elements, and common predictors that have been identified are shown in Figure 2. However, owing to the indications for PPI or the different study methodologies used at different centers, the details of these predictors remain subject to debate. For instance, the cutoffs for membranous septum length or implantation depth may differ based on the study institution, leading to inconsistent findings. In the future, researchers could effectively reduce the risk of the need for postoperative PPI in patients who undergo TAVR by refining the implantation technique or updating the design of next-generation THVs without increasing the risk of perivalvular leakage or coronary artery obstruction.

## 4 The types and effects of conduction abnormalities after TAVR

### 4.1 Impact of new-onset left bundle branch conduction block after TAVR

**Figure 3:**
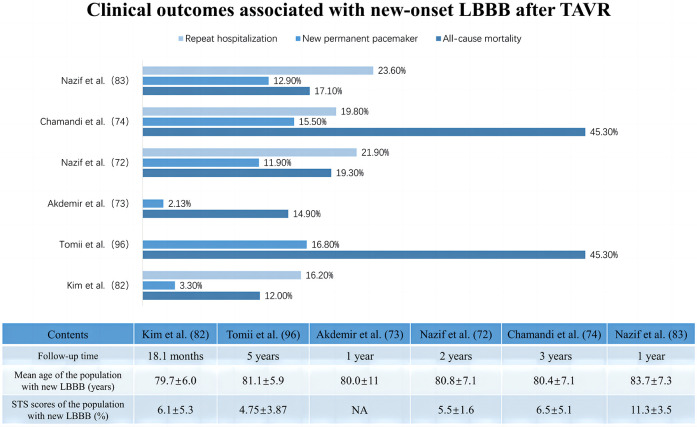
Clinical impact of new onset of left bundle branch block after transcatheter aortic valve replacement.

As the left bundle branch is proximate to the interleaflet triangle between the right coronary and noncoronary cusps, new-onset LBBB is frequently observed after TAVR. However, its clinical impact shows heterogeneity across studies, influenced by its statistical power, follow-up duration, definition of new-onset LBBB, and patient demographics at various institutions (Table 2) (72–74). Additionally, new-onset LBBB has been found to vary dynamically, with some cases resolving over time, while others may persist or progress to HAVB requiring PPI (80–83). This variability may be related to LBBB pathogenesis, individual aortic root anatomy, extent of tissue damage, and recovery rate (84, 85). Short-term follow-up at the University of Minnesota Medical Center revealed no significant difference in all-cause mortality or the PPI rates at one year between patients with and without new-onset LBBB after TAVR, although the former group had a lower LVEF at one year (51.8 ± 11.2 vs. 57.6 ± 8.3; *p* = 0.002) and higher rates of PPI during the index hospitalization (14.9% vs. 0%; *p* < 0.001) (73). Chamandi et al. also observed that new-onset persistent left bundle branch block (NOP-LBBB) after TAVR was not correlated with increased all-cause mortality, cardiovascular mortality, or heart failure rehospitalization rates but potentially increased the risk of PPI (15.5% vs. 5.4%; adjusted HR: 2.45; 95% CI: 1.37–4.38; *p* = 0.002) and worsened left ventricular function (Δ1.9 ± 0.6% vs. Δ1.4 ± 0.9%; *p* < 0.001 for LVEF over time between groups) (74). Conversely, some studies revealed an association between new-onset LBBB after TAVR and increased all-cause mortality, PPI rates, and cardiac-related hospitalization rates during the follow-up period (86, 87). Intriguingly, Nazif et al., in the PARTNER trial in 2013, reported that new-onset LBBB after TAVR may be independent of 30-day or 1-year all-cause mortality and cardiovascular mortality but was significantly associated with higher repeat hospitalization and PPI rates (*p* = 0.01) and reduced LVEF improvement (*p* = 0.02) at 1 year after discharge (75). However, the findings of their later PARTNER II trial in 2019 indicated that new-onset LBBB after TAVR not only correlated with more repeat hospitalization, more PPI, and declining left ventricular function during the 2-year period after discharge but was also linked to increased all-cause mortality (adjusted HR: 1.98; 95% CI: 1.33–2.96; *p* < 0.001) and cardiovascular mortality (adjusted HR: 2.66; 95% CI: 1.67–4.24; *p* < 0.001) over two years (72). Figure 3 summarizes the clinical impact of new-onset LBBB after TAVR.

**Table 2:** Prognostic impact of different conduction abnormalities and permanent pacemaker implantation after TAVR.

Type of conduction abnormalities	Reference	Year	Region	Centers	Sample size	Type of study	Inclusion period	Type of THV implanted	Primary outcome	Trial number
New LBBB	Nazif, T. M. et al. (75)	2014	The United States of America	26	1151	Randomized controlled trial	April 2007–September 2010	Edwards Sapien Valve	New LBBB was not associated with death, repeat hospitalization, stroke, or myocardial infarction at 1 year, but was associated with a higher rate of PPI and failure of LVEF to improve.	NCT00530894
	Chamandi, C. et al. (74)	2019	The United States of America, Italy	9	1,020	Retrospective study	May 2007–February 2015	Edwards Sapien Valve, Medtronic Core Valve	NOP-LBBB was not associated with a higher mortality or heart failure rehospitalization, but increased the risk of PPI and negatively impacted LVEF over time.	NA
	Nazif, T. M. et al. (72)	2019	The United States of America	57	1,179	Randomized controlled trial	March 2011–January 2016	Edwards Sapien Valve	New LBBB was associated with adverse clinical outcomes at 2 years, including all-cause and cardiovascular mortality. rehospitalization, new PPI, and worsened left ventricular systolic function.	NCT01314313, NCT03222128
	Akdemir, B. & Roukoz, H. (73)	2020	The United States of America	1	151	Prospective study	March 2012–June 2015	Edwards Sapien Valve, Medtronic Core Valve, Lotus Valve	New LBBB after TAVR was associated with a higher risk of PPI during the index hospitalization but not after discharge.	NA
New PPI	Nazif, T. M. et al. (67)	2015	The United States of America	26	1,973	Randomized controlled trial	April 2007–September 2010	Edwards Sapien Valve	New PPI was associated with a longer duration of hospitalization and higher rates of repeat hospitalization and mortality or repeat hospitalization at 1 year.	NCT00530894
	Chamandi, C. et al. (76)	2018	Canada, Italy, Spain	9	1,629	Prospective study	May 2007–February 2015	Edwards Sapien Valve, Medtronic Core Valve	PPI post-TAVR was associated with an increased risk of heart failure rehospitalization and lack of LVEF improvement, but not mortality	NA
	Natanzon, S. S. et al. (77)	2022	Israel	1	1,239	Retrospective study	2008–2019	Edwards Sapien Valve, Medtronic Core Valve	Patients who underwent new PPI had worse combined outcome of death and heart failure hospitalizations	NA
	Tomii, D. et al. (78)	2022	Switzerland	NA	2,370	Randomized controlled trial	February 2011–May 2025	Balloon-expandable Valve, Self-expandable Valve, Mechanically expandable Valve	New PPI conferred a nonsignificant trend about an increased risk of 5-year all-cause mortality.	NCT01368250
Other arrhythmias	Amat-Santos, I. J. et al. (79)	2012	Canada	1	138	Prospective study	May 2007–May 2011	Edwards Sapien Valve	NOAF was associated with a higher rate of stroke/systemic embolism, but not a higher mortality, at 30 days and at 1-year follow-up.	NA
	Cresse, S. et al. (54)	2019	The United States of America	1	386	Retrospective study	April 2008 and June 2017	NA	RBBB was associated with delayed progression to complete heart block and need for pacemaker implantation after TAVR.	NA

LBBB has long been recognized as a risk factor for mortality in the general population and in patients with various cardiovascular diseases. Most studies have revealed that the increased mortality risk in patients with LBBB may be attributed to induced ventricular dyssynchronous contractions, septal motion abnormalities, myocardial fibrotic microscopic remodeling, or mitral regurgitation, which leads to left ventricular dysfunction and ultimately to adverse cardiovascular events such as clinical heart failure (85, 88, 89). For patients with new-onset LBBB after TAVR, relevant studies exploring its impact on mortality and perioperative PPI requirements have yielded conflicting results. Compared to resolvable LBBB, new-onset persistent LBBB seems to result in higher PPI rates and limited LVEF improvement, possibly due to its own detrimental mechanisms and progression to HAVB necessitating PPI (75, 90, 91). This highlights the need for further research to substantiate these findings. Importantly, most existing studies have focused on high-risk or surgically untreatable patients who have a greater overall risk of mortality and multiple competing risk factors for death. This population's characteristics could obscure the realistic clinical impact of new-onset LBBB after TAVR (72).

### 4.2 Impact of the high degree of atrioventricular block and permanent pacemaker implantation after TAVR

Relative to LBBB, HAVB after TAVR is often closely linked to the need for PPI, with approximately 15% of patients developing HAVB within 30 days after TAVR and subsequently requiring PPI (92). Cardiac conduction abnormalities after TAVR are generally transient and influenced by the diversity of patient anatomy, the type of valve implanted, and the depth of implantation (93). Persistent HAVB has been proposed as one of the definitive indications for PPI (94). In a prospective study, Hochstadt et al. defined HAVB (second-degree or complete AVB), any symptomatic bradycardia (SB), and LBBB with HV > 65 ms as the main indications for needing PPI after TAVR (95). A similar study concluded that late second- or third-degree AVB inability to subside, sinus node dysfunction, and symptomatic bradycardia were indicated to require PPI after TAVR (74). It has even been suggested that persistent HAVB lasting more than 24 h is a recognized adaptation for PPI after TAVR (77). While the criteria for PPI after TAVR vary across institutions, most researchers suggest that the indications for PPI be strictly limited to those described in the international guidelines posed by the ACCF/AHA/HRS, which advocate for PPI in patients who present with persistent HAVB or sinus node dysfunction accompanied by symptomatic bradycardia after TAVR but not for those with isolated new-onset LBBB (96–98).

**Figure 4:**
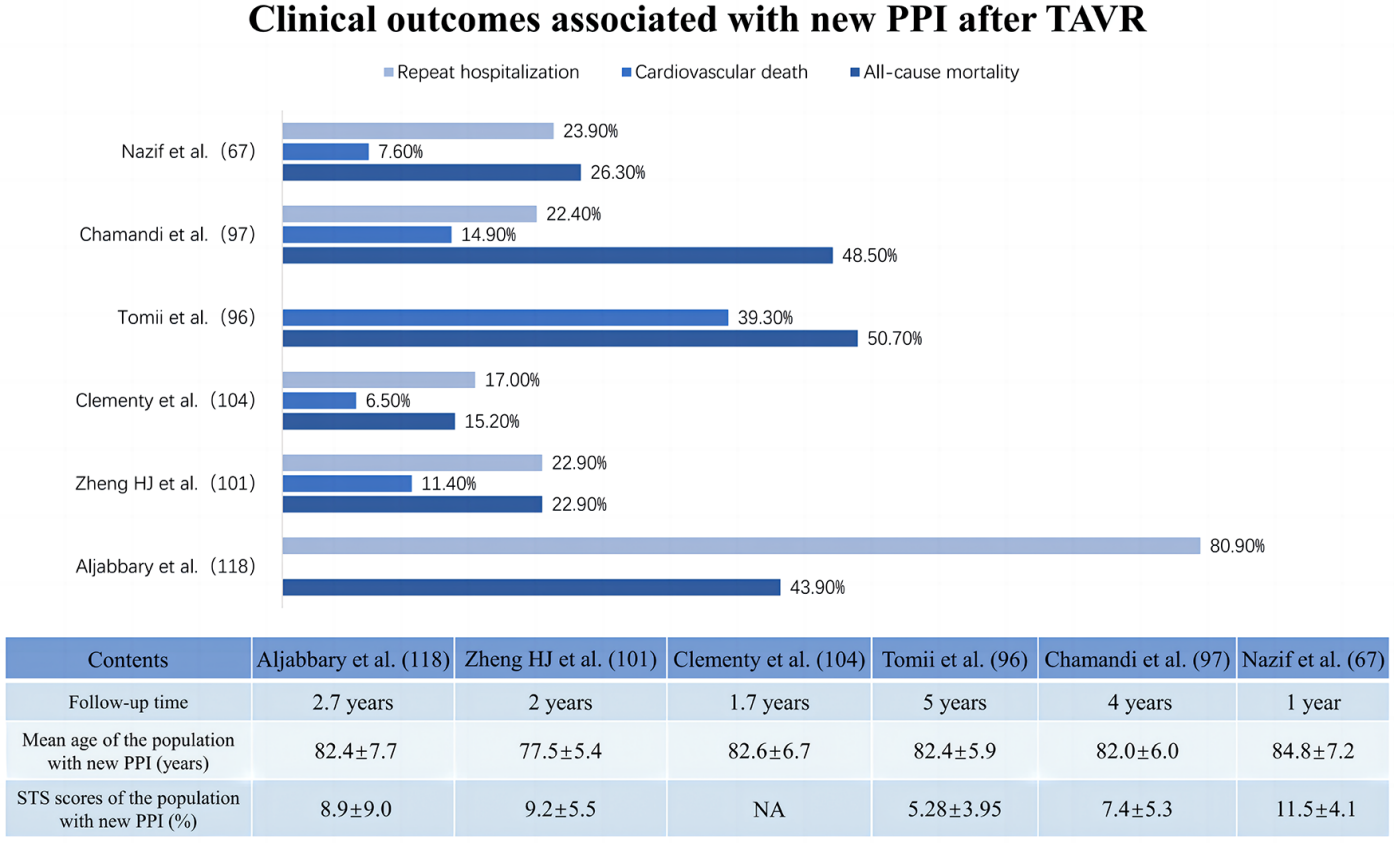
Clinical impact of new permanent pacemaker implantation after transcatheter aortic valve replacement.

Most studies have focused on the prognostic impact of HAVB-associated permanent pacemaker implantation rather than HAVB alone. The available results on the clinical impacts of PPI after TAVR show a paradoxical trend. Some studies have shown that post-TAVR PPI may not be associated with increased adverse events, including all-cause mortality and cardiovascular death (78). A multicenter cohort study concerning the long-term outcomes of PPI after TAVR reported similar overall or cardiovascular mortality rates at 4 years between patients with and without PPI after TAVR but a significantly greater rehospitalization risk for heart failure (22.4% vs. 16.1%; adjusted HR: 1.42; 95% CI: 1.06–1.89; *p* = 0.019) and a trend toward lower LVEF (*p* = 0.051 for between-group LVEF change) in PPI patients, especially those with preexisting LV dysfunction (*p* = 0.005 for between-group LVEF change) (76). These findings are echoed in a meta-analysis by Mohananey et al. (99). Conversely, a meta-analysis enrolling 83,082 patients reported that increased long-term all-cause mortality (RR: 1.18; 95% CI: 1.09–1.28; *p* < 0.0001), heightened risk of heart failure rehospitalization (RR: 1.42; 95% CI: 1.18–1.71; *p* = 0.0002) and reduced LVEF improvement (95% CI: −4.88–2.26; *p* < 0.00001) were associated with PPI after TAVR (100). Nazif et al. (67) and Natanzon et al. (77) also concluded that patients who underwent PPI after TAVR seemed to have a clearly higher risk of 1-year mortality and heart failure hospitalization or repeat hospitalization than patients who did not undergo PPI. Figure 4 summarizes the clinical impact of PPI after TAVR.

It is unclear whether new PPI after TAVR is associated with poorer prognostic outcomes or simply indicative of patients at elevated risk. While the PPI effectively protects against severe atrioventricular block and life-threatening insidious chronic arrhythmias (101), early evidence indicates a potential link between persistent high-frequency right ventricular pacing (RVP) and an increased risk of cardiovascular death and heart failure rehospitalization (102). Bruno's multicenter study with a 6-month follow-up on patients who underwent PPI after TAVR revealed that those with RVP ≥ 40% had significantly higher risks of cardiovascular death and hospitalization for heart failure than patients with RVP < 40% (103). The underlying detrimental mechanism might involve the slow and myocardial depolarization caused by RVP, with asynchronous electrical and mechanical activity between ventricles. This asynchrony of electrical and mechanical activities results in delayed left ventricular activation compared to that of the right ventricle, potentially leading to left ventricular systolic dysfunction, diminished ejection fraction, and even negative left ventricular remodeling (104). Long-term complications associated with permanent pacemakers, especially infections, as well as the possibility that left ventricular electromechanical activation dyssynchrony due to right ventricular pacing may induce or exacerbate mitral and tricuspid regurgitation, which may all be reasons why PPI is associated with increased mortality rates (105–107).

### 4.3 Impact of other arrhythmias

New-onset atrial fibrillation (NOAF) is also a relatively common arrhythmia after TAVR, with prevalence rates ranging between 6.8% and 32% (79, 108–110). Some researchers have suggested that patients with NOAF after TAVR have increased risks of death, heart failure hospitalization, stroke, and hemorrhage compared to patients without AF or with preexisting AF (109, 110). Nontransfemoral artery access was considered the most powerful influencing factor for NOAF. However, Amat-Santos et al. reported no significant differences in overall or cardiac mortality between patients with and without NOAF after TAVR, although NOAF patients had higher incidences of stroke (13.6% vs. 3.2%) and systemic embolic events (15.9% vs. 3.2%) (79). Although the relationship between NOAF and mortality after TAVR is not consistently clear, NOAF frequently emerges as a predictor of adverse outcomes such as hemorrhage and ischemic cardiovascular events (108). Patients who develop NOAF after TAVR are typically older, have more comorbidities, poorer cardiac function, and larger left atrial volumes and are more likely to have undergone balloon dilatation or nonfemoral arterial access, particularly via the transapical route (109, 111). This mechanism is probably ascribed to pericardial and epicardial disruption (112) or an inflammatory response such as trauma (113). Interestingly, compared with single-chamber ventricular pacing (VVI), preoperative permanent pacemaker implantation for TAVR might reduce the incidence of postoperative NOAF, with single-chamber atrial pacing (AAI) or dual-chamber atrial pacing (DDD) associated with a lower AF risk, possibly due to synchronized atrial and ventricular pacing preventing atrial remodeling and inhibiting ectopic atrial foci that lead to AF (114). According to the Class IIa recommendations of the AHA/ACC/HRS guidelines on oral anticoagulants and amiodarone, antithrombotic management is critical for patients with NOAF after TAVR, emphasizing the need for tailored strategies to ensure safer outcomes (115). Notably, antithrombotic regimens for patients treated with TAVR across institutions and specific guidelines for the clinical application of NOAF management after TAVR are lacking. The potential benefits of alternatives such as left auricular occlusion in patients unsuitable for standard anticoagulant treatment remain to be further explored (109).

Although less attention has been given in existing studies to other arrhythmic events after TAVR, such as right bundle branch block (RBBB), left anterior fascicular block (LAFB), intraventricular conduction delay (IVCD), and severe bradycardia, these occurrences are clinically significant. As highlighted by Wang et al. in a case report, other new-onset conduction abnormalities beyond AVB or LBBB after TAVR pose risks of potentially progressing to severe conduction blocks or indicating significant damage to the cardiac conduction system, prompting the development of adverse events (116). Cresse et al., in a single-center retrospective study, reported that patients with new-onset RBBB after TAVR had a higher incidence of complete atrioventricular block (CHB) and PPI requirement than those without new-onset RBBB (OR: 13.2; 95% CI: 4.18–41.70; *p* < 0.0001) (54). Moreover, anatomic studies suggest that owing to early anatomic separation of the right and left bundle branches, the proximal branch of the right bundle branch may emerge first to the left of the interventricular septum, which is susceptible to valve-related damage (117). Sometimes, only right bundle branch damage can be detected after TAVR but may actually be accompanied by damage to the left bundle branch. Therefore, clinicians should be aware of this phenomenon in practice and prioritize the occurrence of arrhythmic events other than LBBB and AVB. Further research is needed to elucidate the relationship between these arrhythmias and the progression to HAVB or the increased rate of PPI.

## 5 Management of conduction abnormalities after TAVR

With the increasing prevalence of TAVR, cardiac conduction abnormalities following the procedure remain a concerning and persistently addressed complication (118). Telemetric ECG monitoring and temporary pacemaker implantation are widely accepted as clinical management strategies for post-TAVR conduction abnormalities. Telemetric monitoring can swiftly detect abnormal cardiac electrical activities, temporary pacemakers serve a provisional substitution role, and permanent pacemaker implantation is often advocated as a subsequent corrective measure (101). According to a class I recommendation of the 2013 ESC Guidelines on cardiac pacing and cardiac resynchronization therapy, clinical observation of up to 7 days is required for patients who develop high or complete AV block after TAVR to assess whether the rhythm disturbance is transient and resolves. However, in case of complete AV block with low rate of escape rhythm this observation period can be shortened since remission is unlikely. If apparent bradyarrhythmia does not resolve during the recommended observation period after TAVR, permanent cardiac pacing should be performed (96). Although the 2013 ESC guidelines address pacing after TAVR, there is no in-depth discussion of this topic. Later, in the 2018 ACC/AHA/HRS Guideline on the Evaluation and Management of Patients With Bradycardia and Cardiac Conduction Delay, regarding the management of conduction abnormalities after TAVR, the Class I recommendation is that permanent pacing before discharge is suggested for patients who develop new AV block with symptoms or hemodynamic instability after TAVR. The Class IIa recommendation is that careful monitoring of bradycardia should be recommended in patients with new persistent bundle branch block after TAVR. The Class IIb recommendation is that PPM implantation should be considered in patients who develop a new persistent LBBB after TAVR (94).

Recently, some studies have found that patients without LBBB, RBBB, or first-degree AVB and those with atrial fibrillation but no BBB or bradycardia after TAVR were less likely to develop delayed HAVB, thus they have been recommended to remove the temporary pacemaker early in the postoperative period and to avoid prolonged telemetric monitoring so as to minimize complications related to temporary pacemaker leads and to shorten hospitalization time. However, patients with conduction abnormalities such as LBBB or RBBB after TAVR are at a high risk of progressing to HAVB requiring PPI, and continuous telemetric ECG monitoring is essential (119, 120). Despite these approaches, uncertainties persist regarding the practical application of electrophysiological studies, the appropriate duration for telemetric monitoring, regulations surrounding temporary pacemakers, and decisions related to the selection and optimal timing of PPI, all of which require further comprehensive research to improve patient prognosis and refine management protocols after TAVR.

### 5.1 Telemetric ECG monitoring

Despite the current debates over the optimal duration for post-TAVR telemetric ECG monitoring, studies have recommended monitoring for up to 72 h to detect the occurrence of late malignant arrhythmic events (121). Gils et al. suggested extended monitoring for at least 6 days for patients with normal baseline conduction but persistent postoperative QRS prolongation (122). Some researchers advocate for up to 30 days of monitoring for a comprehensive assessment of arrhythmic events and timely intervention (81). Toggweiler et al., however, proposed that patients without any conduction abnormalities or with a stable ECG for 48 h after TAVR may be considered for earlier and safer hospital discharge (119). The debate on the appropriate duration of telemetric ECG monitoring after TAVR continues. Regardless of the presence of baseline or new conduction abnormalities after TAVR, routine daily 12-lead ECGs during hospitalization are recommended. Ultimately, the management of post-TAVR patients and decisions on hospital stay durations should prioritize safety without compromising care quality.

### 5.2 Pacemaker-related management

With the increasing incidence of post-TAVR conduction abnormalities, there is growing interest in research on the prognostic impact of permanent pacemaker implantation in TAVR patients. Despite its inherently low operative risk, the PPI tends to have less favorable clinical outcomes, particularly in patients with preexisting left ventricular dysfunction (11, 123). Some researchers have suggested the adoption of specialized algorithms to minimize the number of VPs, the exploration of additional physiological pacing patterns that are appropriate for patients with persistent CHB after TAVR, or the use of advanced pacing strategies such as the use of dual chamber pacemakers (DDDRs) and replacement strategies such as cardiac resynchronization therapy (CRT). These approaches might enhance overall outcomes for patients who are receiving PPI after TAVR (124, 125). However, while DDDR and CRT may reduce the risk of mortality and improve heart failure outcomes in patients with LBBB or severe LV systolic dysfunction, their benefits are not as pronounced in patients with preserved LV systolic function (126).

Recently, His-Purkinje conduction system pacing (HPCSP) has emerged as a more physiological pacing method that involves directly stimulating the His bundle or left bundle branch area. This technique drives stimulation down the physiological conduction pathway and promotes ventricular electrical activity synchronization, encompassing His bundle pacing (HBP) and left bundle branch pacing (LBBP) (42, 127). Compared to RVP, the HPCSP has been proven to be effective in shortening QRS intervals and improving cardiac function in TAVR patients (128). Specifically, HBP has been found to be potentially related to the normalization of QRS intervals and the progressive reestablishment of normal ventricular activation patterns in patients with new-onset LBBB after TAVR (129). However, compared with RVP and LBBP, HBP poses greater challenges in terms of implantation difficulty and pacing parameters. More studies are needed to thoroughly assess the benefits of HBP relative to conventional RVP in the future.

A minimalist approach including left ventricular guidewire rapid pacing, local anesthesia with conscious sedation, radial approach for secondary arterial access and echocardiographically guided vascular access, among others, has also recently been proposed to reduce the invasiveness and shorten the duration of the TAVR procedure for early discharge (130). Among these, left ventricular guidewire rapid pacing is considered a safer and more reliable alternative to temporary pacing, with lower complication rates than implantation of a temporary pacemaker in the right ventricle (131). There is a proposed algorithm for temporary pacing in TAVR proposed by some researchers, left ventricular guidewire rapid pacing is recommended as a priority for all patients with a previously implanted permanent pacemaker or without the history of PPI and high-risk factors such as baseline RBBB. However, for patients without a prior history of PPI but with a high risk for conduction disturbance, an upfront right ventricular pacing strategy via internal jugular (IJ) vein access may be safer because of the potential for CHB to be induced by left ventricular guidewire placement before establishing the left ventricular pacing circuit (132). Overall, left ventricular guidewire rapid pacing can be performed routinely in most cases, and upfront IJ vein pacing wire placement is considered a reasonable approach even in patients with high-risk features, but additional large randomized trials are needed to estimate the safety and efficacy of LV pacing as well as the cost-effectiveness of LV pacing in TAVR.

### 5.3 Electrophysiology (ESP)

According to the latest ESC guidelines for cardiac pacing and cardiac resynchronization therapy, patients with new-onset LBBB after TAVR, exhibiting QRS intervals ≥150 ms or PR intervals ≥240 ms, should undergo ambulatory electrocardiographic monitoring or EPS for early risk stratification. This approach aids in reducing the duration of long-term monitoring while providing valuable insights for decision-making and timing of PPI (80, 97). However, the optimal timing for EPS and the best cutoff value for HV intervals remain uncertain. Various HV interval thresholds, such as ≥55 ms (133), ≥65 ms (134), ≥70 ms (135), and the most commonly used ≥75 ms (121), are considered significant predictors of AVB after TAVR. According to the guidelines, placing a temporary pacemaker for 24–48 h in all patients who develop LBBB after TAVR is recommended, with EPS suggested for those exhibiting sustained dynamic changes in PR or QRS intervals within 48 h (101, 136). Although delayed damage to the conduction system may not be detectable on early ECGs, prophylactic implantation of permanent pacemakers in these patients is not universally endorsed.

### 5.4 Cusp-overlapping projection (COP)

Historically, the standard 3-cusp coplanar projection, in which the 3 coronary cusps are in the same plane at the time of release of the THV, has been the preferred surgical viewing projection for balloon-expandable THV, and the cusp-overlapping projection (COP) is a new technique based on a modification of the classical implantation technique (CIT). Moreover, the COP technique has the potential to become the gold standard for surgically observing projections of self-expanding THVs. Some studies have shown that compared with CIT, the COP technique significantly reduces the risk of PPI after the implantation of self-expanding valves for TAVR without increasing the incidence of adverse outcome events (137, 138). The COP technique offers equal safety and efficacy even in balloon-expandable and mechanically expandable TAVR procedures. Stephan et al. showed that the application of COP significantly reduced the incidence of LBBB after TAVR with repositionable mechanically expandable valves and the rate of PPI after TAVR with balloon-expandable valves compared with the standard TCC prediction (139). These potential advantages of the COP technique in reducing the occurrence of conduction disorders after TAVR may be related to its tendency to provide insights into the right anterior oblique and caudal projections, allowing the aortic annulus and delivery system to be in similar planes, which reduces parallax in the catheterization bands, better displays the left ventricular outflow tract and the aortic root, and provides relative release from the membranous septum, allowing for more precise measurements, better contiguous alignment, and greater THV implantation (138, 140). Although COP technology may provide potential benefits in reducing the PPI rate, data on the impact of COP technology on clinical outcomes are still scarce, and studies with larger samples are needed to assess its efficacy and safety.

## 6 Conclusion

The persistence of cardiac conduction abnormalities after TAVR remains a significant barrier to its broader application in younger and lower-risk cohorts. Addressing this challenge necessitates comprehensive preoperative assessments encompassing anatomical, electrocardiographic, and surgical risk factors. The intraoperative use of COP technique or vigilance in prosthesis width and implantation depth selection is crucial for better controlling valve positioning and release and minimizing tissue damage surrounding the LVOT, especially in severely calcified aortic valves. Postprocedure, more standardized ambulatory ECG monitoring, short-term temporary pacemaker implementation, timely introduction of electrophysiologic studies, and other measures are vital for those patients who are at high risk of developing HAVB requiring PPI, with careful monitoring of the PR, QRS, and HV intervals to optimally time permanent pacemaker implantation. Alternatively, strategies that may improve the long-term prognosis of patients, such as DDD or HPCSP, may be chosen. Overall, with the expanding indications for TAVR, researchers must continually identify the anatomical locations of the conduction system adjacent to the region of procedural manipulation and learn more about the predictors, developmental trends, prognostic consequences, and optimal management strategies of new conduction abnormalities after TAVR.
